# Structural and functional basis of low-affinity SAM/SAH-binding in the conserved MTase of the multi-segmented Alongshan virus distantly related to canonical unsegmented flaviviruses

**DOI:** 10.1371/journal.ppat.1011694

**Published:** 2023-10-13

**Authors:** Hua Chen, Sheng Lin, Fanli Yang, Zimin Chen, Liyan Guo, Jing Yang, Xi Lin, Lingling Wang, Yanping Duan, Ao Wen, Xindan Zhang, Yushan Dai, Keqing Yin, Xin Yuan, Chongzhang Yu, Yarong He, Bin He, Yu Cao, Haohao Dong, Jian Li, Qi Zhao, Quan Liu, Guangwen Lu

**Affiliations:** 1 Department of Emergency Medicine, State Key Laboratory of Biotherapy, West China Hospital, Sichuan University, Chengdu, Sichuan, China; 2 Department of Wound Repair and Rehabilitation Medicine, State Key Laboratory of Trauma, Burns and Combined Injury, Daping Hospital, Army Medical University, Chongqing, China; 3 Disaster Medicine Center, West China Hospital, Sichuan University, Chengdu, Sichuan, China; 4 State Key Laboratory of Biotherapy and Cancer Center, National Clinical Research Center for Geriatrics, West China Hospital, Sichuan University, Chengdu, Sichuan, China; 5 School of Basic Medical Sciences, Chengdu University, Chengdu, Sichuan, China; 6 College of Food and Biological Engineering, Chengdu University, Chengdu, Sichuan, China; 7 Center of Infectious diseases and Pathogen Biology, Key Laboratory of Organ Regeneration and Transplantation of the Ministry of Education, The First Hospital of Jilin University, State Key Laboratory of Zoonotic Diseases, Changchun, Jilin, China; Fundación Instituto Leloir-CONICET, ARGENTINA

## Abstract

Alongshan virus (ALSV), a newly discovered member of unclassified *Flaviviridae* family, is able to infect humans. ALSV has a multi-segmented genome organization and is evolutionarily distant from canonical mono-segmented flaviviruses. The virus-encoded methyltransferase (MTase) plays an important role in viral replication. Here we show that ALSV MTase readily binds S-adenosyl-L-methionine (SAM) and S-adenosyl-L-homocysteine (SAH) but exhibits significantly lower affinities than canonical flaviviral MTases. Structures of ALSV MTase in the free and SAM/SAH-bound forms reveal that the viral enzyme possesses a unique loop-element lining side-wall of the SAM/SAH-binding pocket. While the equivalent loop in flaviviral MTases half-covers SAM/SAH, contributing multiple hydrogen-bond interactions; the pocket-lining loop of ALSV MTase is of short-length and high-flexibility, devoid of any physical contacts with SAM/SAH. Subsequent mutagenesis data further corroborate such structural difference affecting SAM/SAH-binding. Finally, we also report the structure of ALSV MTase bound with sinefungin, an SAM-analogue MTase inhibitor. These data have delineated the basis for the low-affinity interaction between ALSV MTase and SAM/SAH and should inform on antiviral drug design.

## Author summary

The prevalence of ALSV in ticks and domestic animals and the capacity of ALSV to cross species-barriers to infect humans have raised great public health concern. ALSV is featured with a four-segmented genome, with a distant evolutionary relationship to classical unsegmented flaviviruses. The virus encoded MTase, which is essential for viral replication, represents a plausible antiviral target. In this study, we showed that ALSV MTase exhibited significantly lower SAM-/SAH-binding affinity than other flaviviral MTases. By solving crystal structures of the enzyme in the unbound and the SAM/SAH bound states, we further delineated the basis underlying the observed lower binding avidity. Finally, we also reported the MTase-SIN complex structure to facilitate antiviral drug design. Taken together, we have provided solid structural and biochemical evidence to characterize ALSV MTase as a novel low-affinity SAM-/SAH-binding enzyme.

## Introduction

In 2019, a novel tick-borne segmented RNA virus was isolated from the sera of infected patients in northeast China. The virus, designated as Alongshan virus (ALSV), was categorized into the Jingmenvirus group in the unclassified *Flaviviridae* family [1]. The clinical symptoms of ALSV infection were highly similar to those reported in cases of infectious tick-borne encephalitis virus (TBEV, another flavivirus member) infection. The patients commonly suffered from fever and headache. In addition, all the patients had a history of tick bites [1]. Notably, another member of the Jingmenvirus group [2–7], Jingmen tick virus (JMTV), was also confirmed to be able to infect humans [8]. The emergence and spread of these newly identified viral pathogens have posed a potential threat to human health [9]. Structural and functional characterization of the key proteins encoded by these viruses is, therefore, of great significance in terms of studying the viral pathogenesis, identifying feasible antiviral targets, and finally preventing the viral infections.

The phylogenetic analyses of the genome sequences have shown that members of the Jingmenvirus group are more related to the viruses in the *Flavivirus* genus than those of the other genera (e.g., *Hepacivirus* genus, *Pestivirus* genus and *Pegivirus* genus) in the *Flaviviridae* family [1]. The canonical flaviviruses of the *Flavivirus* genus, as exemplified by dengue virus (DENV), Zika virus (ZIKV), Japanese encephalitis virus (JEV), and TBEV, all contain a single-stranded positive-sense RNA genome, which possesses a single long open reading frame (ORF) [10]. This ORF encodes a polyprotein that would be subsequently processed, by the viral and cellular proteases, into multiple structural (capsid [C], pre-membrane [prM] or membrane [M], and envelope [E]) and non-structural (NS) proteins (NS1, NS2A, NS2B, NS3, NS4A, NS4B, and NS5) [11,12]. In contrast, both ALSV and JMTV (as typical Jingmenvirus group members) are featured with a segmented genome that is composed of four single-stranded positive-sense RNA segments [1,2]. Among them, segment 2 and segment 4 encode viral structural proteins VP1, VP2, and VP3 of evolutionarily unknown origin; segment 1 and segment 3 encode the viral non-structural proteins of NSP1 and NSP2, which are individually homologous to the NS5 and NS2B-NS3 non-structural proteins of the canonical unsegmented flaviviruses [1,2,13].

ALSV NSP1 is a typical flavivirus NS5-like protein. It carries two essential enzymatic activities: the methyltransferase (MTase) and the RNA-dependent RNA polymerase (RdRp). With an indispensable role in viral replication [14], the flavivirus NS5 protein is long considered to be a feasible target for antiviral-drug design and development [15–17]. The flaviviral NS5-MTase functions to catalyze the methylation of viral RNA cap [18,19]. It can sequentially methylate the RNA cap at the N7 position of the guanine and the ribose 2’-O position of the first replicated adenosine [20]. Such methylation process would sequentially generate cap0 structure (featured as ^7Me^GpppA…) and cap1 structure (featured as ^7Me^GpppA_2’-O-Me_…) using S-adenosyl-L-methionine (SAM) as the methyl donor and generating S-adenosyl-L-homocysteine (SAH) as the reaction byproduct [20,21]. This methylation step is crucial for viral genome stability, efficient translation, and evasion of the host immune response [19]. Structurally, flavivirus MTase is composed of a central MTase core with a typical Rossmann fold and the flanking N- and C-terminal extensions which cradle the core on one side [16,19,21–23]. SAM and SAH bind in a cleft within the core domain. The solved structures of SAM/SAH-bound MTases from different flaviviruses have revealed a highly conserved SAM/SAH-binding pocket [16,19,24]. While canonical flavivirus MTases have been well studied both structurally and functionally, MTases from any Jingmenvirus group members remain uncharacterized.

In this study, we report the atomic crystal structures of ALSV H3 strain MTase in both the free and the SAM/SAH-bound forms. Via detailed structural analyses and comparison, the similarities and differences between ALSV MTase and the canonical flavivirus MTases are specified. By focusing on the SAM/SAH-binding pocket, the basis of ligand engagement by ALSV MTase is delineated. Based on the solution binding data, we show that ALSV MTase is a low-affinity SAM/SAH-binding enzyme *in vitro*. Via mutagenesis, we demonstrate that the difference in the pocket-lining β2/β3 interloop between ALSV and DENV3 MTases is a key factor leading to their variant binding affinities when engaging SAM. Finally, we also report the complex structure of ALSV MTase bound with SIN (sinefungin, an analogue of the methyl-donor SAM) and characterize their interactions in solution, which should inform on structure-based antiviral drug design, optimization, and modification in the future.

## Results

### Low-affinity binding of ALSV MTase to SAM and SAH

**Fig 1:**
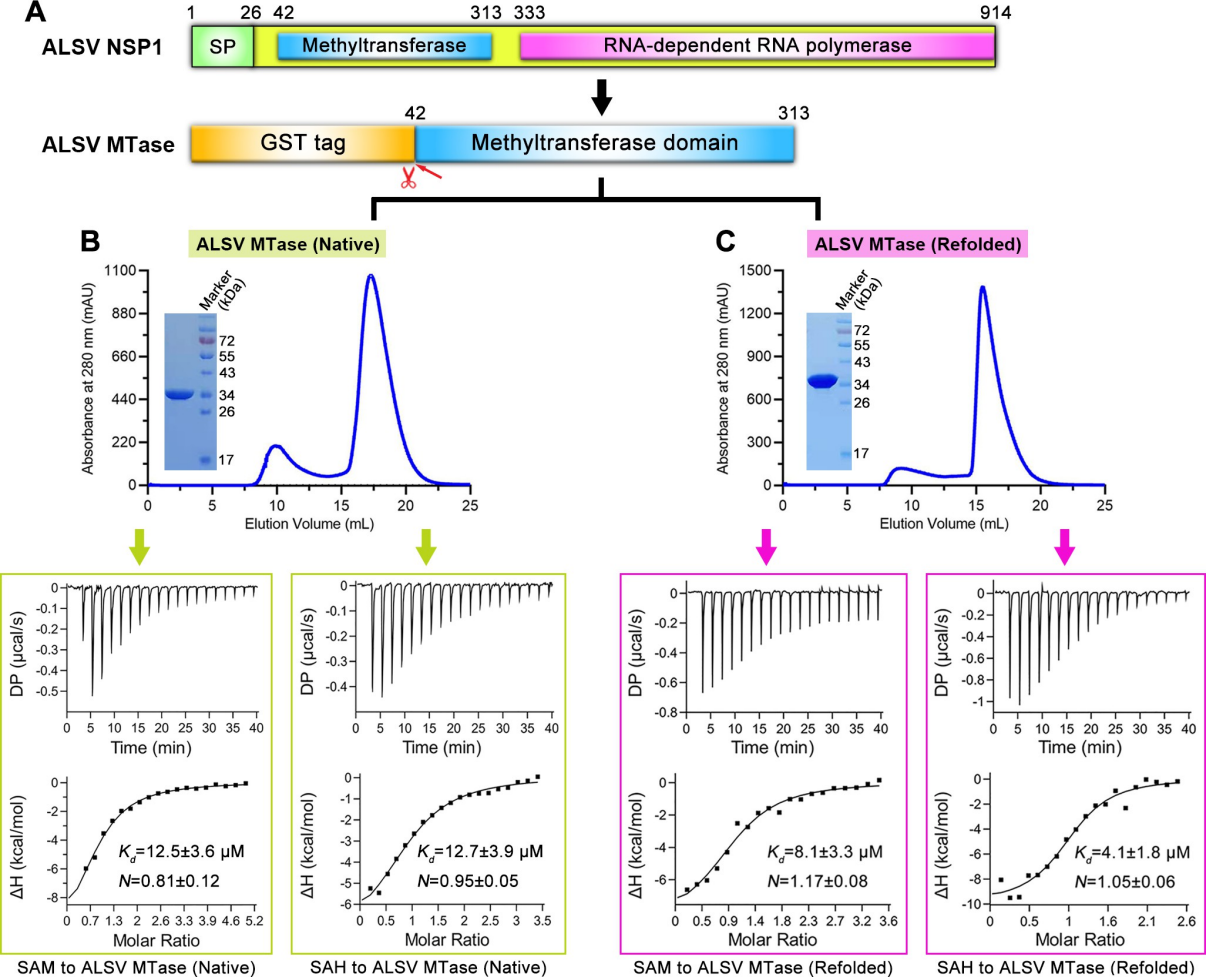
Biochemical characterization of ALSV MTase. (**A**) A schematic view of the protein-engineering strategy used to yield ALSV MTase. The putative signal peptide (SP), the methyltransferase domain, and the RNA-dependent RNA polymerase domain are individually marked with the boundary-residue numbers. ALSV MTase is initially expressed as a GST-fusion protein, which is then enzymatically cleaved to remove the GST tag. (**B** and **C**) The upper panel, solution behavior of native (B) or refolded (C) ALSV MTase protein on a Superdex 200 Increase 10/300 GL column. The inset figure shows the SDS-PAGE analyses of the pooled samples. The bottom panel, affinity determination between native (B) or refolded (C) ALSV MTase and SAM or SAH using ITC.

The genomic segment 1 of ALSV encodes a nonstructural protein of 914 amino acids. The resultant NSP1 protein contains a predicted signal peptide or a transmembrane region at the N-terminus (residues 1–26), an MTase domain in the middle (residues 42–313), and an RdRp domain at the C-terminus (residues 333–914) (**Fig 1A**). Although typical MTase motifs could be identified in the ALSV enzyme, MTases of ALSV and the canonical unsegmented flaviviruses shared low sequence identities, ranging from 17.74%-19.92% (**S1 Table**). To learn the biochemical features of ALSV MTase, we expressed the MTase-domain protein in *E*. *coli* and purified the enzyme to high purity and homogeneity via gel-filtration chromatography (**Fig 1B**). On a calibrated Superdex 200 Increase column, ALSV MTase was eluted around 16.9 mL, indicating that the protein existed mainly as a monomer in solution.

We further characterized the binding between ALSV MTase and SAM (the substrate of methyl-donor) as well as SAH (the product molecule after catalysis) using isothermal titration calorimetry (ITC). The binding affinities for SAM and SAH were determined to be 12.5 ± 3.6 μM and 12.7 ± 3.9 μM, respectively (**Fig 1B**). In comparison to previously reported affinity-values of SAM/SAH bound to the MTases of the canonical flaviviruses (as exemplified by DENV3 [25]), the values observed for ALSV MTase represented substantially lower binding affinities.

To ascertain such affinity difference, we further prepared the DENV3 MTase protein and investigated the solution binding behaviors between DENV3 MTase and SAM via ITC. It is notable that almost all the MTase proteins from canonical flaviviruses would have simultaneously captured SAM/SAH in their SAM/SAH-binding pockets during expression in *E*. *coli* [26–29]. Accordingly, marked differences in both the affinity-value and the *N*-value (an index of the SAM/MTase binding stoichiometry) could be expected for the MTase protein directly purified from *E*. *coli* (with pre-bound SAM) and the MTase protein after a denaturing-refolding cycle (pre-bound SAM was removed). For clarity, we designated the MTase protein obtained directly from *E*. *coli* as native MTase and the protein after the denaturing and refolding treatments as refolded MTase hereafter. The native and refolded MTase proteins from both DENV3 (**S1A and S1B Fig**) and ALSV (**Fig 1B and 1C**) were subsequently prepared and then used in parallel for ITC analyses.

For DENV3 MTase, the affinity of SAM binding to the native protein was determined to be 1.40 ± 0.95 μM. As expected, we observed a very low *N* index, featuring with a value of 0.03 ± 0.04. For refolded DENV3 MTase, however, the affinity was determined to be 156 ± 8.11 nM and the *N* index to be 0.81 ± 0.002, respectively (**S1C and S1D Fig**). The marked differences observed between the native and the refolded proteins were in good accordance with previous studies [25], demonstrating that the SAM/SAH-binding pocket of DENV3 MTase was indeed pre-occupied during expression.

For ALSV MTase, the binding affinities of SAM and SAH to the refolded enzyme were determined to be 8.1 ± 3.3 μM and 4.1 ± 1.8 μM, respectively (**Fig 1C**). In comparison to the native protein, these values represented only a slight/moderate increase in the binding affinity. The denaturing-refolding cycle therefore did not significantly affect the ligand binding activity of ALSV MTase, forming a sharp contrast to that observed with the DENV3 protein. For the *N* indices, the values were calculated to be 0.81 ± 0.12 and 0.95 ± 0.05 for native protein with SAM and SAH and 1.17 ± 0.08 and 1.05 ± 0.06 for refolded protein with SAM and SAH, respectively (**Fig 1B and 1C**). The results highlight an overall stoichiometric *N* index of around 1 for both the native and the refolded proteins, indicating that 1. SAM/SAH binds to ALSV MTase in a 1:1 molar ratio and 2. the SAM/SAH-binding pocket in ALSV MTase is largely unoccupied during expression. It is also notable that, in comparison to refolded DENV3 MTase, ALSV MTase binds SAM with an approximate 80-fold lower affinity for native ALSV MTase and about 52-fold lower affinity for refolded ALSV MTase, respectively.

We then performed a comparative methyltransferase activity assay using the ALSV MTase protein (native), the DENV3 MTase protein, and the ALSV NSP1 protein that contains both MTase and RdRp domains (**S2A Fig**). An nsp10/nsp14 complex protein derived from severe acute respiratory syndrome coronavirus 2 (SARS-CoV-2), prepared as previously described [30], was used as a positive control. Echoing the above ITC data, the results clearly showed that, in comparison to DENV3 MTase, ALSV MTase exhibited weaker enzymatic activity (**S2B Fig)**. In addition, we also observed that the ALSV NSP1 protein, with both the MTase and the RdRp domains, showed enhanced MTase activity when compared to the single MTase-domain protein (**S2B Fig)**. The results indicated a possible stabilizing allosteric effect of the NSP1 RdRp domain on the MTase domain, increasing its enzymatic activity to promote viral replication. It is also noteworthy that similar phenomenon of inter-domain connections for enhanced MTase activity has been observed in the non-structural protein nsp14 of SARS-CoV-2 [31].

To learn the structural features leading to low-affinity binding between ALSV MTase and SAM/SAH, we subsequently solved, via X-ray crystallography, the structures of ALSV MTase in both the unbound and the SAM/SAH-bound forms (see Results below).

### Structure of ALSV MTase in the unbound form

**Fig 2:**
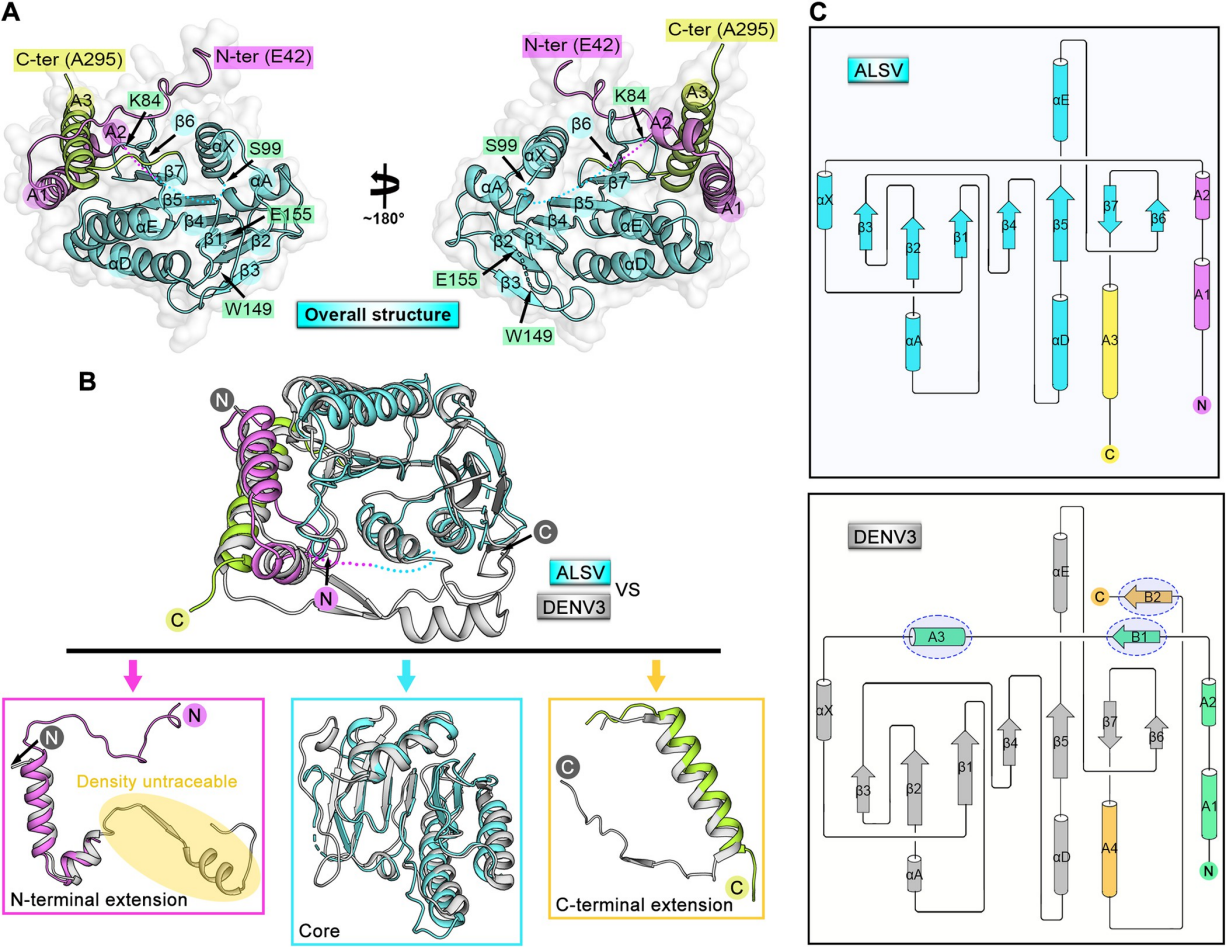
Structure of ALSV MTase. (**A**) Overall structure of ALSV MTase with three subdomains being presented in violet (N-terminal extension region), cyan (core region) and lemon (C-terminal extension region) colors, respectively. The secondary structural elements are labeled with nomenclatures described for DENV2 MTase [22]. The regions that are not visible in the structure are indicated with dashed lines. The terminal residues that are density-traceable are also labeled. (**B**) The upper panel, superposition of the ALSV MTase structure onto the previously reported DENV3 MTase structure (PDB code: 4R8R) [32]. The color scheme for our structure is the same as in (A), and the DENV3 MTase structure is shown in gray. The bottom panel, comparison of the individual ALSV MTase subdomains with those of DENV3 MTase. (**C**) Topological plots of ALSV MTase [shown in upper panel, subdomains are individually colored in violet (N-terminal extension region), cyan (core region) and yellow (C-terminal extension region)] and DENV3 MTase [shown in bottom panel, subdomains are individually colored in green (N-terminal extension region), gray (core region) and orange (C-terminal extension region). Cylinders and arrows represent helices and strands, respectively. Compared with ALSV MTase, the additional secondary structural elements in DENV3 MTase are highlighted by oval boxes.

The crystal structure of apo ALSV MTase was determined using the single-wavelength anomalous dispersion method and finally refined to a resolution of 2.5 Å, with *R*_work_ and *R*_free_ values of 0.209 and 0.258, respectively (**S2 Table**). Clear electron densities could be observed for residues E42-K84, S99-W149 and E155-A295. Amino acids Y85-P98, E150-E154 and D296-R313, however, were unable to be traced due to high flexibility. Overall, the ALSV enzyme has a globular fold, which can be further divided into three subdomains (the N-terminal and C-terminal extension regions and the core region) (**Fig 2A**). The N-terminal extension region consists of an extended loop followed by a helix-turn-helix motif (helices A1 and A2). The core region folds into a seven stranded β-sheet (strands β1-β7) surrounded by four α helices (helices αX, αA, αD and αE), which reveals the general αβα-Rossmann-fold [22,23]. The C-terminal extension region consists mainly of an α-helix (helix A3).

The overall structure of ALSV MTase is similar to those of the MTase proteins from canonical unsegmented flaviviruses. Using the previously reported structure of DENV3 MTase (PDB code: 4R8R) as a representative [32], superimposition of the structures revealed that a majority of the secondary structural elements could be well aligned (**Fig 2B**). The root mean square deviation (RMSD) was calculated to be 2.1 Å for 199 equivalent Cαs. The core region of ALSV MTase mostly resembled its DENV3 homologue and therefore featured with an SAM-dependent methyltransferase fold [23]. The N-terminal and C-terminal extension regions, however, showed marked differences between the two enzymes (**Fig 2B and 2C**). For ALSV MTase, it contained a long-extended N-terminal loop, which was not present in DENV3 MTase. In addition, following the helix-turn-helix motif in the N-terminal extension, the ALSV enzyme also featured with a shortened loop that is highly flexible (amino acids Y85-P98 being untraceable in the electron density map). Its DENV3 equivalent, however, was of longer length, folding into an extra α-helix (helix A3) and an additional β-strand (strand B1) in the N-terminal extension region. For the C-terminal extension, ALSV MTase contained a single α-helix (helix A3) whereas DENV3 MTase possessed an α-helix (helix A4) and an additional β-strand (strand B2).

### Structures of ALSV MTase in complex with SAM and SAH

**Fig 3:**
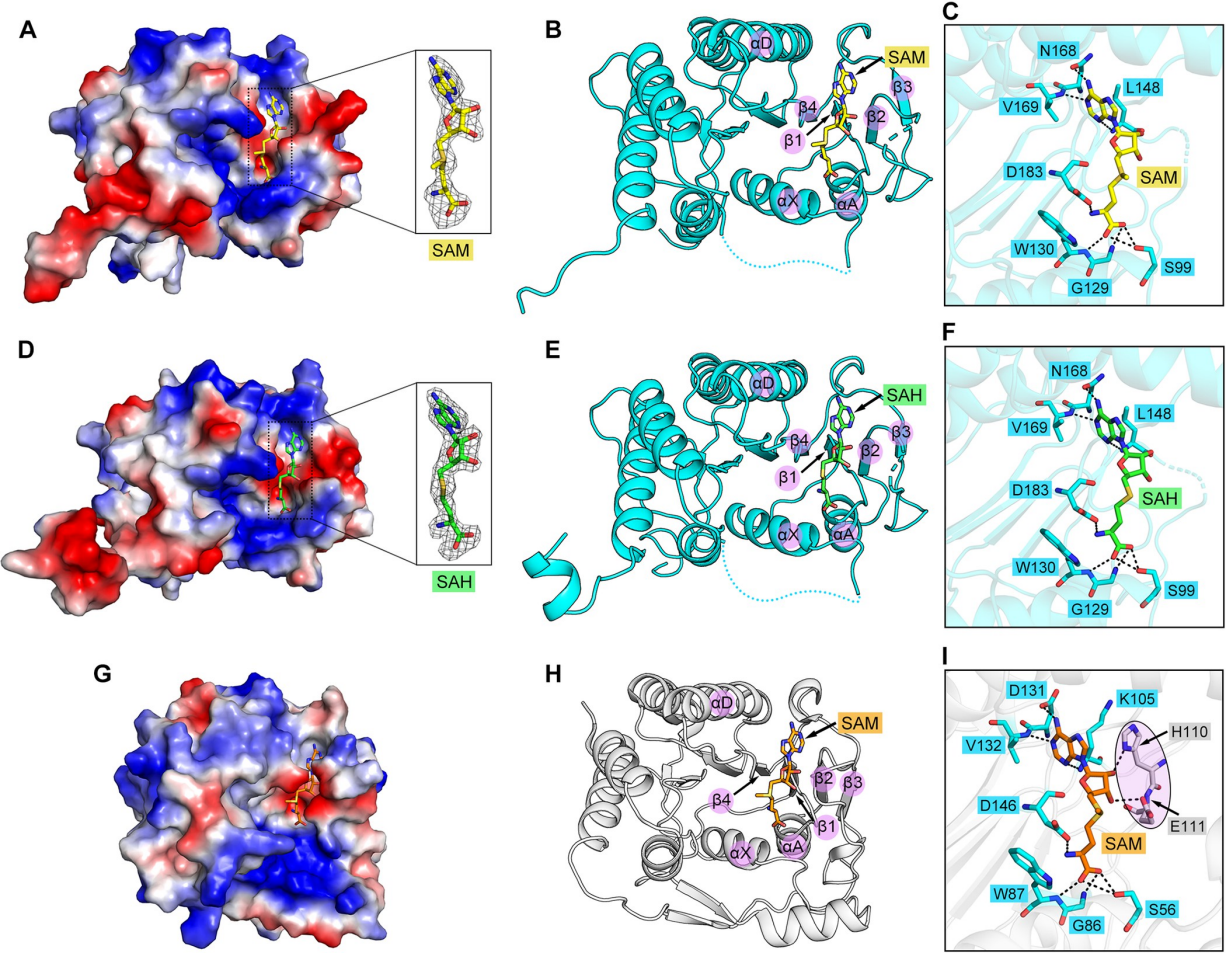
Complex structures of ALSV MTase with SAM and SAH. (**A** and **D**) An electrostatic-surface representation of ALSV MTase in the SAM-bound (A) and SAH-bound (D) forms. The SAM and SAH molecules are shown as yellow and green sticks, respectively. The SAM/SAH-binding pocket is indicated with black dotted rectangle. The bound SAM and SAH molecules, whose electron densities are contoured at 2.8 σ using the |Fo|-|Fc| map, are shown in the black rectangle. (**B** and **E**) A cartoon representation of ALSV MTase in the SAM-bound (B) and SAH-bound (E) forms. Those structural elements involved in the formation of the ligand-binding pocket are labeled. (**C** and **F**) Detailed interactions between ALSV MTase and SAM (C) or SAH (F). Dashed lines indicate hydrogen bonds. (**G**, **H** and **I**) Overview of the SAM-binding pocket in DENV3 MTase (based on PDB code: 5E9Q). (**G**) An electrostatic-surface representation of DENV3 MTase bound with SAM. The SAM molecule is shown as orange sticks. (**H**) A cartoon representation of DENV3 MTase bound with SAM. Those structural elements involved in the formation of the ligand-binding pocket are labeled. (**I**) Detailed interactions between DENV3 MTase and SAM. Dashed lines indicate hydrogen bonds. Those SAM-binding residues in DENV3 MTase that are equivalent to those responsible for ALSV-MTase/SAM interactions are shown as cyan sticks, while the other two residues in DENV3 MTase that form additional hydrogen bonds with SAM are highlighted and shown as gray sticks.

The crystals of ALSV MTase bound with SAM and SAH were individually obtained by co-crystallization at a protein/ligand molar ratio of 1:3. The structure of ALSV MTase in complex with SAM was solved at 2.3 Å, with *R*_work_ and *R*_free_ values of 0.192 and 0.241, respectively (**S2 Table**). The structure of ALSV MTase in complex with SAH was solved at 2.1 Å, with *R*_work_ and *R*_free_ values of 0.210 and 0.236, respectively (**S2 Table**). In each case, clear electron densities for the bound ligands were observed (**Fig 3A and 3D**), which enabled us to define, at the atomic level, the SAM/SAH-binding pocket.

In the complex structures, the SAM/SAH molecule fits into a conserved pocket located at the edge of the ALSV MTase core region (**Fig 3A and 3D**). The pocket is surrounded by helices αX, αA and αD, strands β1-β4, and several adjacent loops (**Fig 3B and 3E**). The SAM/SAH molecule lies down in the pocket, within which the bound ligand is stabilized by multiple hydrogen-bond interactions. These include residues S99, G129, W130 and D183 of the MTase forming five hydrogen bonds with the methionine/homocysteine group of SAM/SAH, and amino acids L148, N168 and V169 mediating another three hydrogen bonds with the adenosyl group of SAM/SAH (**Fig 3C and 3F**).

### Structural basis for the low affinity binding between ALSV MTase and SAM/SAH

**Fig 4:**
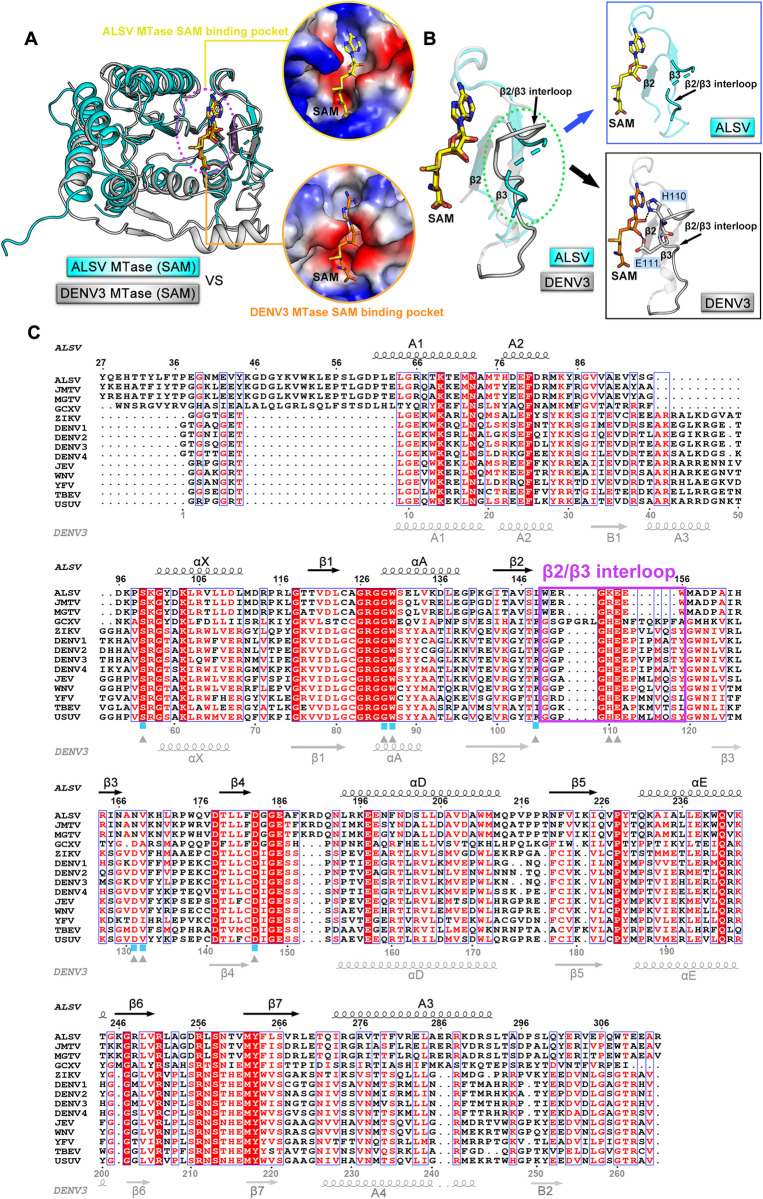
Comparison of the SAM binding pockets between ALSV MTase and the canonical flavivirus MTase. (**A**) Superposition of the ALSV MTase/SAM structure onto the previously reported DENV3 MTase/SAM structure (PDB code: 5E9Q) [33]. Electrostatic-surface representations of SAM binding pockets are shown on the right panel. (**B**) Comparison of the β2/β3 interloops between ALSV MTase and DENV3 MTase. Interactions between β2/β3 interloops and SAM molecules are shown on the right panel. Dashed lines indicate hydrogen bonds. (**C**) Structure-based multiple sequence alignment of methyltransferases from Jingmenvirus group and canonical flaviviruses. The secondary structural elements of ALSV MTase and DENV3 MTase are labeled above and below the sequences, respectively. The residues that interact with SAM molecules are labeled with blue boxes (for ALSV MTase) and grey triangles (for DENV3 MTase). The β2/β3 interloops are highlighted with violet rectangle.

We next selected a previously reported structure of DENV3 MTase bound with SAM (PDB code: 5E9Q) [33] to compare with our structure of ALSV MTase in complex with SAM solved in this study. Similar to that observed in the ALSV structure, the DENV enzyme build a deep pocket, with helices αX, αA and αD, strands β1-β4, and the adjacent loops, for SAM binding (**Fig 3G and 3H**). Superimposition of the structures revealed sterically conserved pockets within the core region. In addition, the SAM ligand adopted the same extended conformation, lying down in the pocket (**Fig 4A**). Therefore, ALSV MTase shared a conserved SAM/SAH-binding pocket with unsegmented flavivirus MTases despite of their low sequence identities.

We further scrutinized the binding pockets in the two structures and compared the SAM-binding interactions in detail between the ALSV and DENV3 MTases. Those inter-molecule hydrogen bonds identified in the ALSV structure were also observed to form in the DENV3 structure (**Fig 3I**). In addition, DENV3 MTase further provided two additional hydrogen bonds to stabilize the bound SAM molecule: one by residue H110 and the other by residue E111 to interact with the adenosyl group of SAM (**Figs 3I and 4B**). These two amino acids resided in the loop connecting strands β2 and β3 (hereafter designated as the β2/β3 interloop). It is notable that this β2/β3 interloop lines the side-wall of the SAM/SAH-binding pocket. In ALSV MTase, this loop is of short length and high flexibility, devoid of any stabilizing contacts with the SAM/SAH ligand. In DENV3 MTase, however, the loop is of longer length such that it is able to half-cover SAM/SAH, thereby properly positioning residues H110 and E111 to provide additional hydrogen-bond interactions to stabilize the bound ligand (**Fig 4B and 4C**). The marked difference in the β2/β3 interloop between ALSV MTase and DENV3 MTase seems to coincide well with the affinity difference observed between the two enzymes when interacting with SAM.

**Fig 5:**
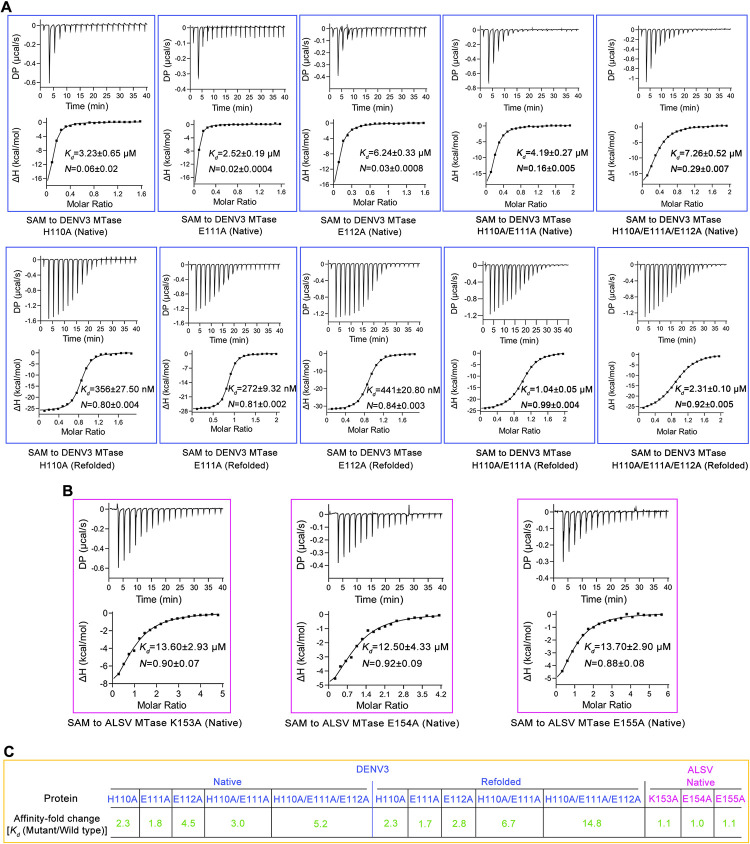
Affinity determination between MTase mutants and SAM using ITC. (**A**) Binding of SAM to native or refolded DENV3 MTase mutants (H110A, E111A, E112A, H110A/E111A or H110A/E111A/E112A). (**B**) Binding of SAM to native ALSV MTase mutants (K153A, E154A or E155A). (**C**) Summary of affinity-fold change between mutant MTase and wild-type MTase [*K*_d_ (mutant/wild type)]. The *K*_d_ values for wild-type DENV3 MTase are shown in **S1C Fig** (native protein) and **S1D Fig** (refolded protein). The *K*_d_ value for wild-type ALSV MTase is shown in **Fig 1B** (native protein).

Guided by the structural observations, we further designed a series of ALSV and DENV3 MTase mutants in which key residues in the pocket-lining β2/β3 interloop were substituted with alanine. The mutations included H110A, E111A and E112A in DENV3 MTase and K153A, E154A and E155A in ALSV MTase. The mutant proteins were then prepared (**S3 Fig**) and analyzed for their SAM-binding capacities via ITC (**Fig 5A–5C**). Because the DENV3 protein would automatically capture SAM during expression, both the native and the refolded proteins were prepared for the DENV3 MTase mutants. As expected, the mutations of H110A and E111A in DENV3 MTase both led to decreased binding affinities (**Fig 5A and 5C**). In addition, the substitution of E112 with alanine also apparently affected SAM binding. This residue did not directly interact with SAM. Nevertheless, the side-chain of E112 formed two hydrogen bonds with G107 and S128 (**S4 Fig**), which could in turn stabilize the conformation of the β2/β3 interloop for SAM engagement.

We further introduced double and triple amino-acid mutations into the β2/β3 interloop of DENV3 MTase to investigate if these residues might have a synergistic effect in mediating SAM binding. The double-mutant (H110A/E111A) and triple-mutant (H110A/E111A/E112A) proteins (both the native protein and the refolded protein) were firstly prepared to high purity and homogeneity (**S3 Fig**). Subsequently, a quantitatively comparative study on the SAM-binding affinity were conducted using ITC with these mutant enzymes. The determined affinities for the refolded double-mutant and triple-mutant proteins were 1.04 ± 0.05 μM and 2.31 ± 0.10 μM, respectively (**Fig 5A and 5C**). These values represented ~7- and ~15- fold decrease in affinity when compared to that of the refolded wild-type protein. The results indicated that residues H110, E111 and E112 showed a synergistic effect when interacting with SAM, which in turn demonstrated that the β2/β3 interloop of DENV3 MTase indeed played an important role in SAM binding. In contrast, residue substitutions in the β2/β3 interloop of ALSV MTase did not affect its binding to SAM (**Fig 5B and 5C**), echoing our structural observation that this loop was devoid of any inter-molecule contacts with SAM.

### Structure of ALSV MTase in complex with SIN

**Fig 6:**
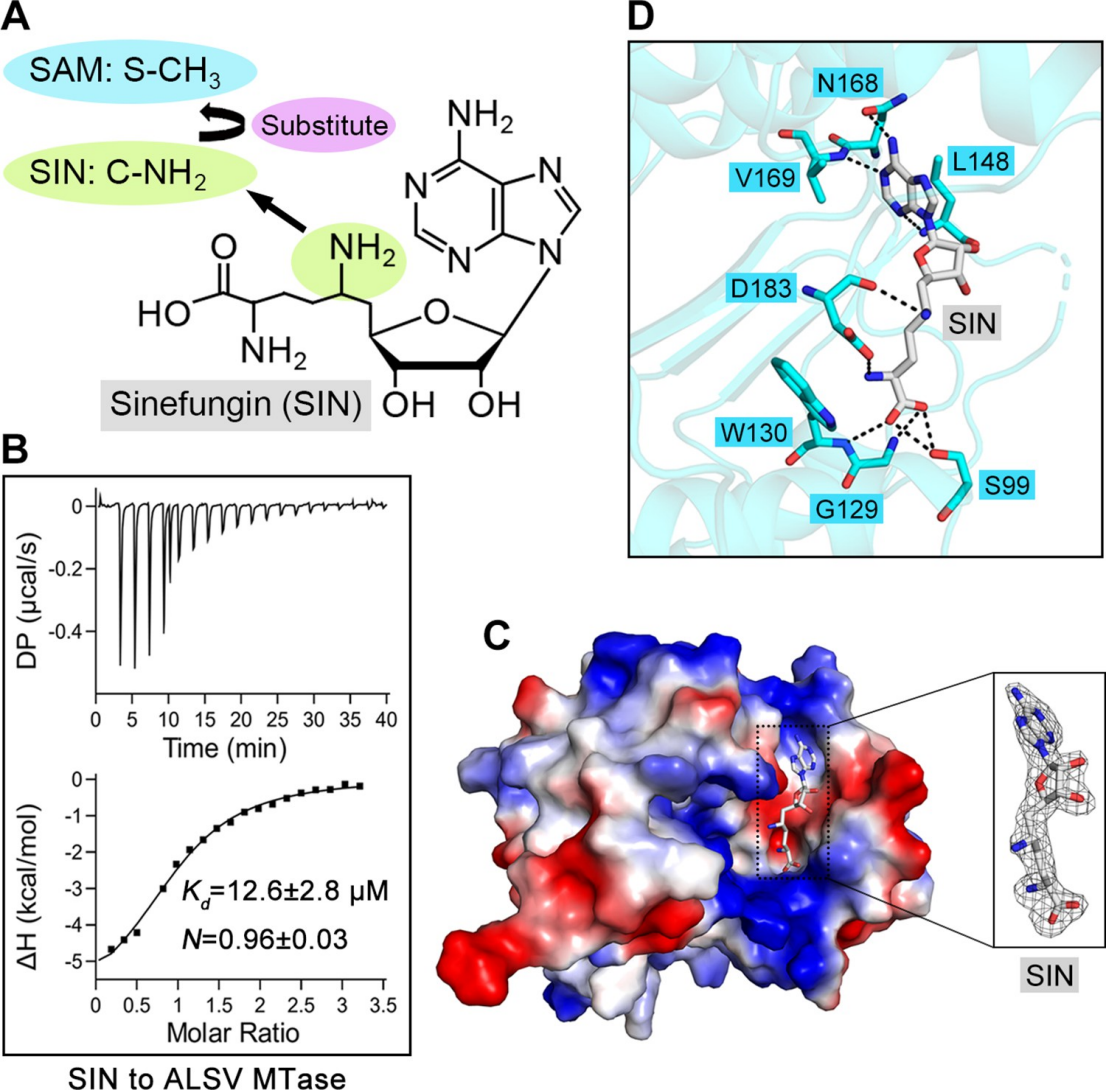
Binding capacity of SIN towards ALSV MTase and the molecular basis of complex formation. (**A**) The chemical formula of sinefungin (SIN), which includes a C-NH_2_ group to substitute the S-CH_3_ group of methyl donor SAM. (**B**) Affinity determination between ALSV MTase and SIN using ITC. (**C**) The overall electrostatic-surface representation of ALSV MTase in the SIN-bound form. The bound SIN molecule, whose electron density is contoured at 2.8 σ using the |Fo|-|Fc| map, is shown in the black rectangle. (**D**) Detailed interactions between ALSV MTase and SIN. Dashed lines indicate hydrogen bonds.

Sinefungin (SIN), an analogue of the methyl-donor SAM, is originally isolated from *Streptomyces griseoleus* as a potential antifungal antibiotic [34]. It contains a C-NH_2_ group to replace the S-CH_3_ group of SAM (**Fig 6A**) and has been previously identified as a potent inhibitor of flavivirus MTases [24,25,35,36]. To ascertain if SIN might also be used as a potential inhibitor against ALSV MTase, we first investigated the binding of SIN to the ALSV MTase protein via ITC. As expected, SIN readily binds to the ALSV enzyme, showing an affinity (12.6 ± 2.8 μM) that is similar to those observed with SAM and SAH (**Fig 6B**). We further determined the crystal structure of ALSV MTase in complex with SIN at 2.0 Å resolution. The structure was solved with *R*_work_ and *R*_free_ values of 0.198 and 0.226, respectively (**S2 Table**). The electron density for the bound SIN molecule was clearly observed in the SAM/SAH-binding pocket (**Fig 6C**). The overall structure of ALSV MTase bound with SIN is nearly identical to those of the protein in complex with SAM and SAH, showing an RMSD of 0.292 Å and 0.246 Å, respectively. Similar to SAM/SAH, SIN adopted an extended conformation, lying down in the pocket. Its binding was stabilized via a series of hydrogen-bond interactions, involving the MTase residues S99, G129, W130, L148, N168, V169 and D183 (**Fig 6D**). These interactions were almost the same as those observed with SAM and SAH, demonstrating that SIN shared a conserved binding mode as SAM/SAH when interacting with ALSV MTase. As expected, our *in-vitro* enzymatic assay clearly showed that SIN, as a broad-spectrum viral methyltransferase inhibitor, preserved its inhibitory capacity against the ALSV enzyme (**S2C Fig**).

## Discussion

Within the family of *Flaviviridae*, the arthropod-borne flaviviruses are notorious for their capabilities to infect humans and in some but not rare cases to cause epidemics and pandemics [37–40]. Typical flaviviruses are featured with a single-stranded positive-sense RNA genome. In recent years, the *Flaviviridae* family has been expanded with the Jingmenvirus group members, including ALSV, JMTV, Mogiana tick virus (MGTV) and Guaico Culex virus (GCXV). These viruses are phylogenetically related to flaviviruses but contain a segmented RNA genome. The public significance of the Jingmenviruses is currently drawing more and more attention as accumulating evidence has shown that these viruses can also cause human infections [1,8]. Notably, a retrospective study has shown that at least 86 patients with complete medical records in northeast China are confirmed to have been infected by ALSV [1]. In addition, ALSV is believed to be transmitted by ticks but has also been identified in domestic animals [1,41]. The prevalence of the virus in both ticks and animals and the capacity of the virus to cross species-barriers to infect humans have raised great public health concern. The flavivirus-encoded MTase plays an important role in stabilizing viral genome, promoting efficient translation, and evading immune recognition [19,42] and therefore represents an attractive target for the development of anti-viral drugs [16,17]. In this study, we have reported the structural characteristics and the ligand-binding features of ALSV MTase, which is, to our knowledge, the first MTase in the Jingmenvirus group that has been characterized both structurally and functionally thus far.

In comparison to MTases of the canonical unsegmented flaviviruses, the MTase of ALSV shows very limited sequence identities. Nevertheless, the structure of ALSV MTase is quite similar to its flavivirus homologues, featuring with three subdomains folding into a typical flaviviral MTase fold. In addition, ALSV MTase also shares a conserved SAM/SAH-binding mode. The binding pocket locates sterically at the same position of the core region and the bound ligand adopts the same extended conformation as observed in the canonical flavivirus MTases. Despite of these similarities, ALSV MTase shows a significantly lower binding affinity towards SAM. Such low-affinity binding is further corroborated by our observation that ALSV MTase is barely co-purified with SAM. In contrast, multiple MTases of canonical flaviviruses are always co-purified with SAM [16,19,22,43,44].

Further comparison between our structures and previously reported structures of flaviviral MTases (using the DENV3 structure as a representative) reveals an interesting conformational difference in their β2/β3 interloops lining the sidewall of the SAM/SAH-binding pocket. In ALSV MTase, the β2/β3 interloop is of short length and devoid of any stabilizing contacts with SAM. In DENV3 MTase, however, this pocketing-lining interloop is of longer length and half-covers SAM, contributing multiple hydrogen-bond interactions. In addition, we further show that the observed difference in the β2/β3 interloop coincides well with our subsequent mutagenesis data. These results demonstrate that the marked difference in the pocket-lining β2/β3 interloop between the two enzymes is indeed a key factor leading to their variant binding affinities when engaging SAM. It is notable that within the Jingmenvirus group, the MTases of JMTV and MGTV resemble that of ALSV, possessing a shortened β2/β3 interloop (**Fig 4C**). We therefore believe that the two viral enzymes should also show a low binding affinity towards SAM. In contrast, the β2/β3 interloop of GCXV MTase is much longer than its ALSV homologue (**Fig 4C**). It remains to be investigated how the GCXV enzyme would perform when interacting with SAM.

The canonical flavivirus MTase locates at the N-terminus of nonstructural protein 5 (NS5). Previous studies have reported a set of complex structures of flavivirus MTase bound with RNA cap analogue, RNA substrate and SAM/SAH, elucidating how viral RNA is specifically recognized and methylated [21,22,26–28,43,44]. When these MTase structures of the canonical unsegmented flaviviruses (including ZIKV, DENV2, DENV3, JEV, West Nile virus (WNV), yellow fever virus (YFV), Murray Valley encephalitis virus (MVEV), TBEV, Omsk hemorrhagic fever virus (OHFV), Modoc virus (MODV) and Yokose virus (YOKV) [16,19,27,29,43–49]) are aligned in parallel with the ALSV MTase structure, a large groove featured with strong basic electrostatic surface could be observed in the equivalent region of all MTases (**S5A Fig**). Previous studies have already demonstrated that this large basic patch is responsible for RNA binding [21]. Thus, ALSV MTase likely shares a similar RNA substrate binding groove with canonical flavivirus MTases. The cap binding site is located beside the RNA binding groove (**S5A Fig**). Sterically, the RNA cap structure always binds in-between the N-terminal extension region and the core region. In canonical flavivirus MTases, cap-binding involves residues: K13, L16, N17, M19, F24, S150 and S215 in ZIKV MTase, K14, L17, N18, L20, F25, K29, S150 and S213 in DENV3 MTase, K13, L16, N17, M19, F24, R28, S150 and S215 in MVEV MTase, and K13, L16, N17, C19, F24, R28, S150 and S216 in OHFV MTase (**S5B Fig**). Structure-based multiple sequence alignment shows that these cap-binding residues are also preserved (either the same or substituted with amino acids of similar characteristics) in ALSV MTase (**S5C Fig**), highlighting conserved interactions for RNA cap engagement in the ALSV enzyme. While cap-binding and RNA-engagement in ALSV MTase likely resemble those in the canonical flavivirus MTases, the ALSV SAM/SAH-binding pocket, with low affinity binding and a unique β2/β3 interloop, could represent a novel feature separating Jingmenvirus MTase from the classical flavivirus MTase.

In summary, we have provided both structural and biochemical evidence to characterize ALSV MTase as a novel low-affinity SAM/SAH-binding enzyme in the *Flaviviridae* family. ALSV MTase retains a typical flaviviral MTase fold and possesses a conserved SAM/SAH-binding pocket. Nevertheless, a shortened pocket-lining loop correlates with its low binding affinity towards SAM and SAH. We believe this information should facilitate antiviral drug design in the future.

## Materials and methods

### Gene cloning, protein expression and purification

The coding sequences of ALSV MTase (residues E42-R313, GenBank: AXE71873.1), ALSV NSP1 (residues E42-N914, GenBank: AXE71873.1) and DENV3 MTase (residues S3-D294, GenBank: AY662691.1) were synthesized and sub-cloned into the pGEX-6p-1 vector with *BamH* І and *Xho* І restriction sites, respectively. The ALSV MTase mutants (K153A, E154A and E155A) and DENV3 MTase mutants (H110A, E111A, E112A, H110A/E111A and H110A/E111A/E112A) were generated with a standard two-step PCR-based strategy. All the recombinant plasmids were transformed into *E*. *coli* BL21 (DE3) cells for protein expression. Cells were grown in LB medium supplemented with 100 μg/mL ampicillin at 37°C and induced for protein expression with 400 μM isopropyl-β-D-thiogalactopyranoside (IPTG) at 16°C for about 12 hours. To prepare ALSV MTase selenomethionine derivative protein (designated as Se-Met-derivate ALSV MTase), the cells were cultured in 1 L of M9 minimal medium at 37°C until the OD_600_ reached at 0.5, and then supplied with 50 mg of Se-Met (selenomethionine) as the sole Met source, by additional adding 0.1 g of Lys, Thr, Phe, and 0.05 g of Leu, Ile, and Val, to inhibit endogenous synthesis of Met. Cells were grown in LB medium at 37°C and then induced by 400 μM IPTG at 16°C overnight.

For the protein purification, the *E*. *coli* cells were harvested, lysed by sonication in a re-suspension buffer consisting of 20 mM Tris-HCl (pH 7.5) and 500 mM NaCl, and clarified via centrifugation at 18,000×g for 30 min. Cleared lysate-supernatant was bound to glutathione-Sepharose resin (GE Healthcare) to remove the contaminated proteins, and cleaved by on-column digestion using PreScission Protease (GE Healthcare) to remove the GST tag. The target proteins were then collected and further purified by gel-filtration chromatography in re-suspension buffer using a Superdex 200 Increase 10/300 GL column (GE Healthcare).

To remove pre-bound SAM/SAH that was simultaneously captured by ALSV MTase/DENV3 MTase during protein expression, the protein derived from *E*. *coli* was first denatured using a buffer consisting of 6 M Guanidine-HCl, 50 mM Tris-HCl (pH 8.0), 100 mM NaCl, 10 mM DTT and 10% Glycerol at a final concentration of 10 mg/mL. The denatured protein was then refolded at 4°C by dialyzing against a buffer containing 100 mM Tris-HCl (pH 8.0), 400 mM L-Arg-HCl, 5 mM reduced glutathione and 0.5 mM oxidized glutathione for about 10 hours. Subsequently, the refolded protein was concentrated using an Amicon Stirred Cell concentrator (Merck Millipore) with a 10-KDa cutoff membrane and then adjusted to re-suspension buffer. The protein was then further purified by gel filtration using a Superdex 200 Increase 10/300 GL column (GE Healthcare).

### Crystallization, data collection and structure determination

For crystallization trials, commercial crystallization kits (Molecular Dimensions and Hampton Research) were used for sitting drop vapor diffusion. Normally, 1 μL protein was mixed with 1 μL reservoir solution. The resultant drop was then sealed, equilibrating against 70 μL reservoir solution at 18°C. The diffractable crystals of the native ALSV MTase protein and ALSV MTase selenomethionine derivative protein were both obtained in 0.5 M Lithium sulfate, 0.1 M Tris (pH 8.5), and 25% w/v PEG 3350. Crystals of the ALSV MTase in complex with SAH (Sigma) were obtained in 0.1 M Tris (pH 8.5), and 3.0 M Sodium chloride by co-crystallizing the protein with SAH at a final concentration of 0.3 mM for the protein and 0.9 mM for SAH. In addition, the crystals of ALSV MTase in complex with SAM (Sigma) or SIN (J&K Scientific) were obtained in 0.5 M Lithium sulfate, 0.1 M Tris (pH 8.5), and 25% w/v PEG 3350 by co-crystallizing the protein with SAM or SIN at a final concentration of 0.3 mM for the protein and 0.9 mM for SAM or SIN.

Data collection were conducted at Shanghai Synchrotron Radiation Facility (SSRF) beamline BL18U1 [50]. The collected data were then processed with HKL2000 [51] for indexing, integration, and scaling. The ALSV MTase apo structure was collected at 2.5 Å and solved using single-wavelength anomalous diffraction [52] in Phenix AutoSol module [53] with default parameters. The structure model was further built manually using COOT [54] based on the experimental phase and then refined using Phenix [55].

The structures of the ALSV MTase in complex with SAH, SAM, or SIN were solved by molecular replacement method using Phaser [56] from CCP4 program suite [57], with the structure of ALSV MTase as the search model. For refinement, initial restrained rigid-body refinement was performed using REFMAC5 [58], which was followed by manual rebuilding and adjustment in COOT [54]. The SAM, SAH, or SIN molecules were manually built using COOT based on the simulated annealing omit Fo-Fc maps and were further refined using Phenix [55]. The stereochemical quality of the final model was assessed through the program PROCHECK [59]. Final statistics for data collection and structure refinement are summarized in **S2 Table**. All structural figures were generated using Pymol (http://www.pymol.org).

### ITC (isothermal titration calorimetry) assay

The ITC measurements were performed at 25°C on an ITC200 calorimeter (Malvern) with a reference power of 5 μcal/s and a stirring speed of 750 rpm. Protein samples were prepared in ITC buffer containing 100 mM Tris-HCl (pH 7.5) and 500 mM NaCl. Ligand (SAM and SAH) samples and the inhibitor (SIN) were dissolved in the same ITC buffer as the proteins. SAM, SAH or SIN (500–800 μM) were titrated into the sample cell containing native or refolded ALSV MTase (30–50 μM). SAM (450 μM) was titrated into the sample cell containing native DENV3 MTase (55 μM). SAM (250 μM) was titrated into the sample cell containing refolded DENV3 MTase (30 μM). All samples were centrifuged at 17,000×g for 20 min before titration. Each titration typically involves 19 injections of 2 μL ligand with 4 s durations and 120 s intervals. The data fitting and analyses were performed using the Origin 7.0 software package provided by MicroCal with a single-site model.

### RNA substrate preparation

A Gppp-capped 351-nt RNA was used as substrate for methyltransferase activity. The uncapped RNA was prepared by *in-vitro* transcription using a double-stranded DNA template [5’-CAGTAATACGACTCACTATT-(DNA sequence from GenBank: AY662691.1, the first 351 bp)-3’] and the TranscriptAid T7 high-yield transcription kit (Thermo Scientific). The reaction was performed in a 20-μL mixture containing 1× TranscriptAid reaction buffer, 10 mM nucleoside triphosphates [NTPs], 1 μg template DNA, and 1× TranscriptAid enzyme mix. The reaction was incubated at 37°C for 2 h. Then, DNA was removed by DNase I treatment, and the RNA was purified using GeneJET RNA Purification Kit (Thermo Scientific). The above prepared RNA was capped by vaccinia capping system (New England Biolabs) following the manufacturer’s instructions to form Gppp-capped RNA. During the reaction, SAM was not added to the enzymatic system, so the product RNA was not methylated. Finally, the capped RNA substrate was further purified using GeneJET RNA Purification Kit.

### Methyltransferase activity assay

The methyltransferase (MTase) activity was measured using the MTase-Glo Methyltransferase bioluminescence assay kit (Promega) following the manufacturer’s instructions. The reaction mix containing 20 mM Tris (pH 8.0), 50 mM NaCl, 1 mM EDTA, 3 mM MgCl_2_, 0.1 mg/mL BSA, 1 mM DTT, 3 μM protein [ALSV MTase (native), ALSV NSP1 (native), DENV3 MTase (native), or SARS-CoV-2 nsp10/nsp14 complex (contains N7-MTase activity, prepared as previously described [30])], 20 μM SAM and 0.3 μM Gppp-capped RNA were incubated for 2 h at 37°C. The reagent solution from the kit was then added, and the mixture was further incubated for 30 min at room temperature, before the addition of the detection solution. Luminescence was measured by using a BioTek Synergy H1 microplate reader. The averages and the s.d. of three measurements were plotted as a histogram using GraphPad Prism 6. For the sinefungin (SIN) inhibition assay, 500 μM SIN was added to the reaction mix, and the other steps are the same as in the methyltransferase activity assay.

## Supporting information

S1 Fig Affinity determination between DENV3 MTase and SAM using ITC. (**A** and **B**) Solution behavior of native (A) or refolded (B) DENV3 MTase protein on a Superdex 200 Increase 10/300 GL column. The inset figure shows the SDS-PAGE analyses of the pooled samples. (**C** and **D**) Affinity determination between native (C) or refolded (D) DENV3 MTase and SAM by ITC.
(TIF)

S2 Fig The methyltransferase activity assay for ALSV MTase. (**A**) Solution behavior of ALSV NSP1 (with both the MTase and the RdRp domains) on gel filtration chromatography. The inset figure shows the SDS-PAGE analyses of the pooled samples. (**B**) The methyltransferase activity of ALSV MTase is compared with ALSV NSP1 and DENV3 MTase. The protein of SARS-CoV-2 nsp10/nsp14 complex was used as a positive control. Data represents the mean ± SD of three independent reactions. (**C**) An ALSV MTase inhibition assay conducted in the presence of 500 μM SIN. Data represents the mean ± SD of three independent reactions.
(TIF)

S3 Fig The protein preparations of DENV3 and ALSV MTase mutants used for affinity determination in Fig 5. Solution behaviors of DENV3 and ALSV mutant MTase proteins on a Superdex 200 Increase 10/300 GL column. The inset figure shows the SDS-PAGE analyses of the pooled samples.
(TIF)

S4 Fig The β2/β3 interloop in DENV3 MTase is stabilized by residues G107 and S128. As the dashed lines indicate, the E112 residue on β2/β3 interloop could form two hydrogen bonds with G107 and S128.
(TIF)

S5 Fig Structural comparison between ALSV MTase and representative flaviviral MTases focusing on the cap binding site and the RNA binding groove. (**A**) Crystal structures of ALSV MTase in complex with SAM, ZIKV MTase in complex with SAM and ^7Me^GpppA (PDB code: 5WZ2), DENV2 MTase in complex with SAH and ^7Me^GpppA (PDB code: 2P3O), DENV3 MTase in complex with SAH and ^7Me^GpppAGUUGUU (PDB code: 5DTO), JEV MTase in complex with SAH (PDB code: 4K6M), WNV MTase in complex with SAH (PDB code: 2OY0), YFV MTase in complex with SAH and GpppA (PDB code: 3EVE), MVEV MTase in complex with SAH and ^7Me^Gppp (PDB code: 2PX8), TBEV MTase in complex with SAM (PDB code: 7D6M), OHFV MTase in complex with SAH and ^7Me^GpppA_2’-O-Me_G (PDB code: 7V1E), MODV MTase in complex with SAM (PDB code: 2WA2), and YOKV MTase in complex with SAM (PDB code: 3GCZ). The RNA cap analogues, RNA substrates, and SAM/SAH molecules, if present in the structures, are shown and labeled. Otherwise, the RNA cap binding sites and RNA binding grooves are indicated with arrows. (**B**) Detailed interactions between methyltransferases of flaviviruses and RNA cap analogues. Dashed lines indicate hydrogen bonds. (**C**) Structure-based multiple sequence alignment highlighting the cap binding site. The secondary structural elements of ALSV MTase are labeled above the sequences, and the secondary structural elements of DENV3 MTase are labeled below the sequences. Those amino acids involved in the RNA cap binding are highlighted with blue triangles.
(TIF)

S1 Table Statistics of the sequence identities among MTase proteins from Jingmenvirus group and canonical flaviviruses. (DOCX)

S2 Table Data collection and structure refinement statistics. (DOCX)
