# Supplementary figures and images for: Structural and functional basis of low-affinity SAM/SAH-binding in the conserved MTase of the multi-segmented Alongshan virus distantly related to canonical unsegmented flaviviruses
Source: PLoS Pathog. 2023 Oct 13;19(10):e1011694. doi: 10.1371/journal.ppat.1011694 (PMC10575543; doi:10.1371/journal.ppat.1011694)

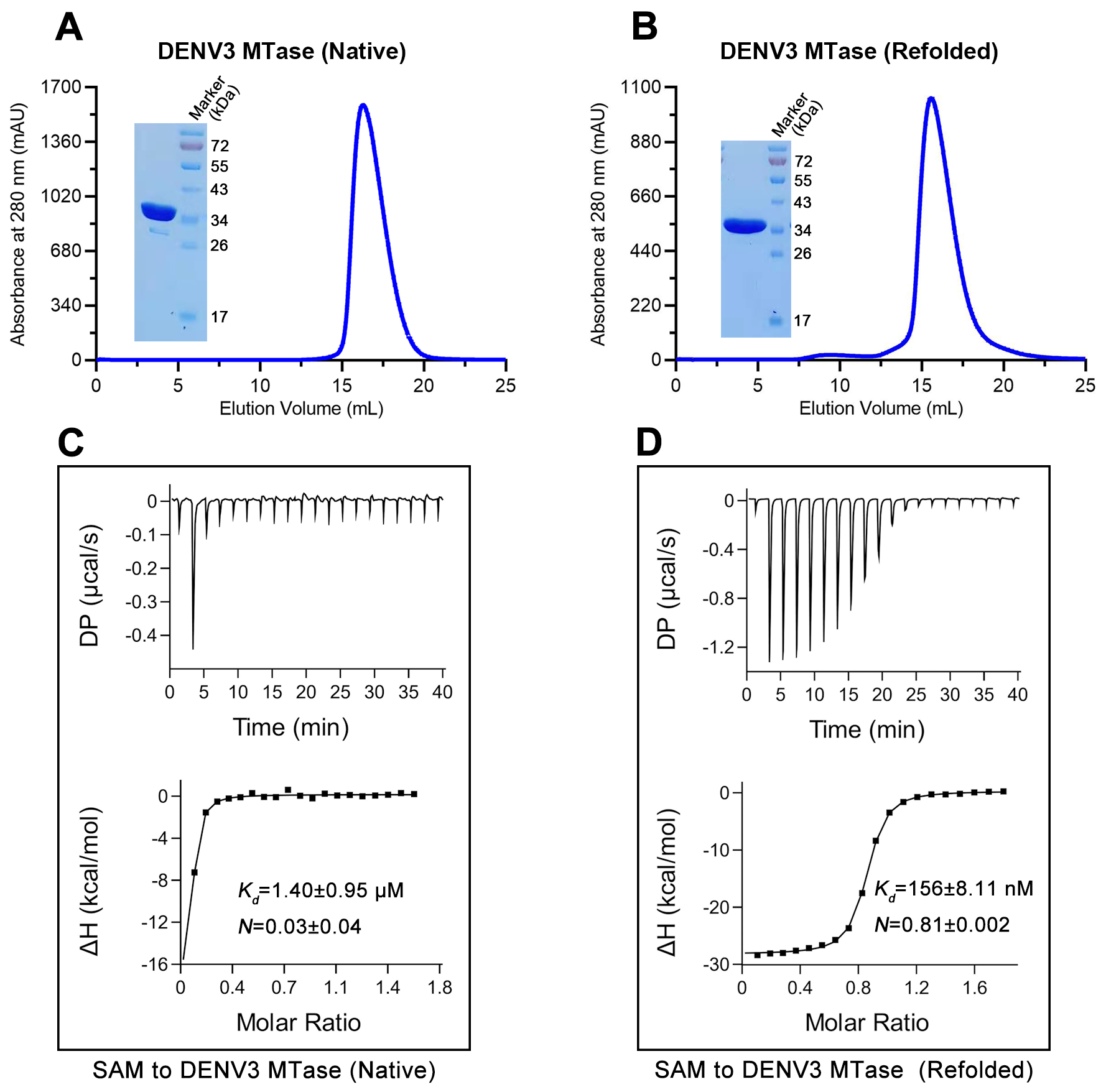

Supplement: S1 Fig — (A and B) Solution behavior of native (A) or refolded (B) DENV3 MTase protein on a Superdex 200 Increase 10/300 GL column. The inset figure shows the SDS-PAGE analyses of the pooled samples. (C and D) Affinity determination between native (C) or refolded (D) DENV3 MTase and SAM by ITC. (TIF) [file ppat.1011694.s001.tif]

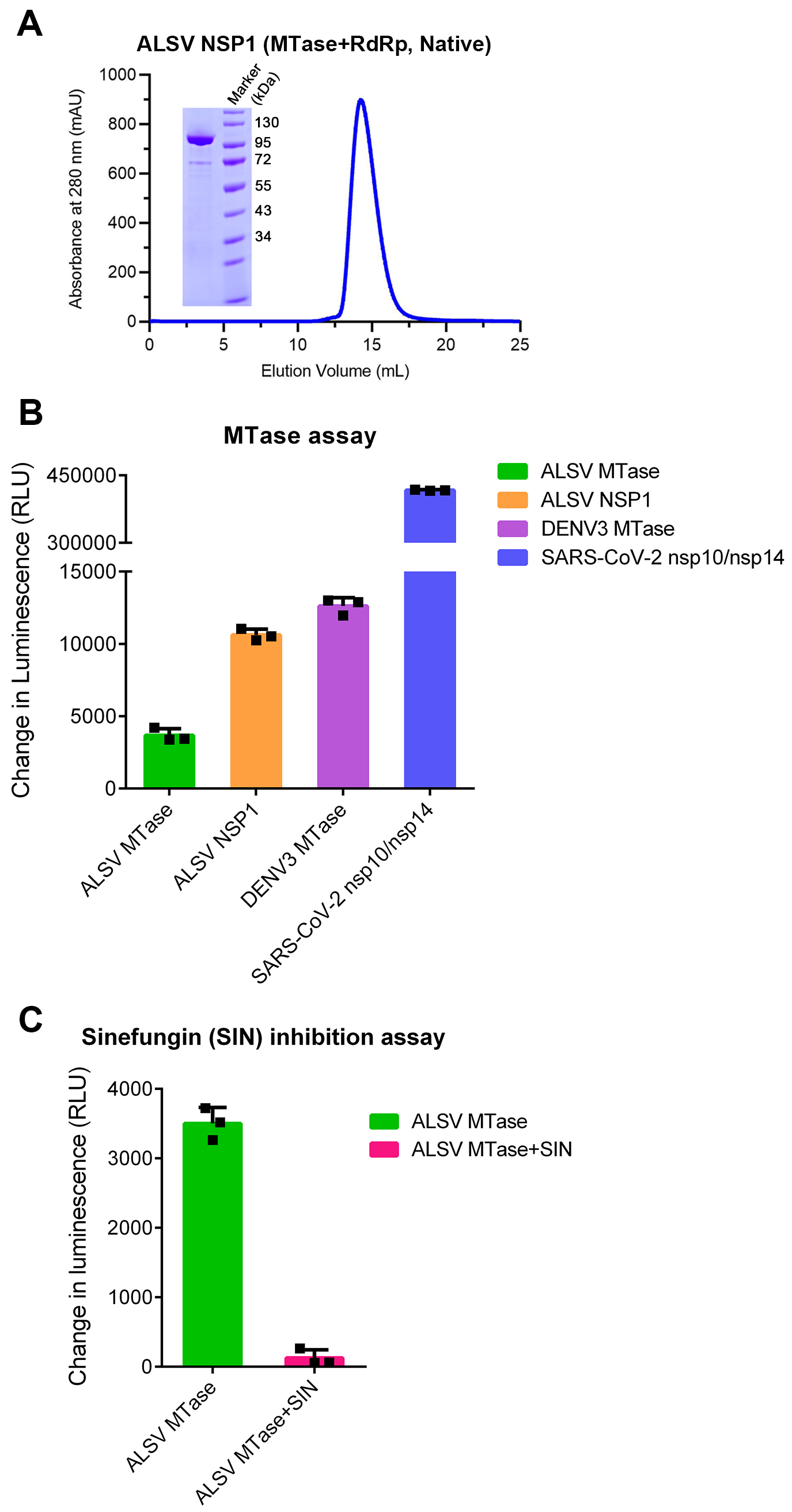

Supplement: S2 Fig — (A) Solution behavior of ALSV NSP1 (with both the MTase and the RdRp domains) on gel filtration chromatography. The inset figure shows the SDS-PAGE analyses of the pooled samples. (B) The methyltransferase activity of ALSV MTase is compared with ALSV NSP1 and DENV3 MTase. The protein of SARS-CoV-2 nsp10/nsp14 complex was used as a positive control. Data represents the mean ± SD of three independent reactions. (C) An ALSV MTase inhibition assay conducted in the presence of 500 μM SIN. Data represents the mean ± SD of three independent reactions. (TIF) [file ppat.1011694.s002.tif]

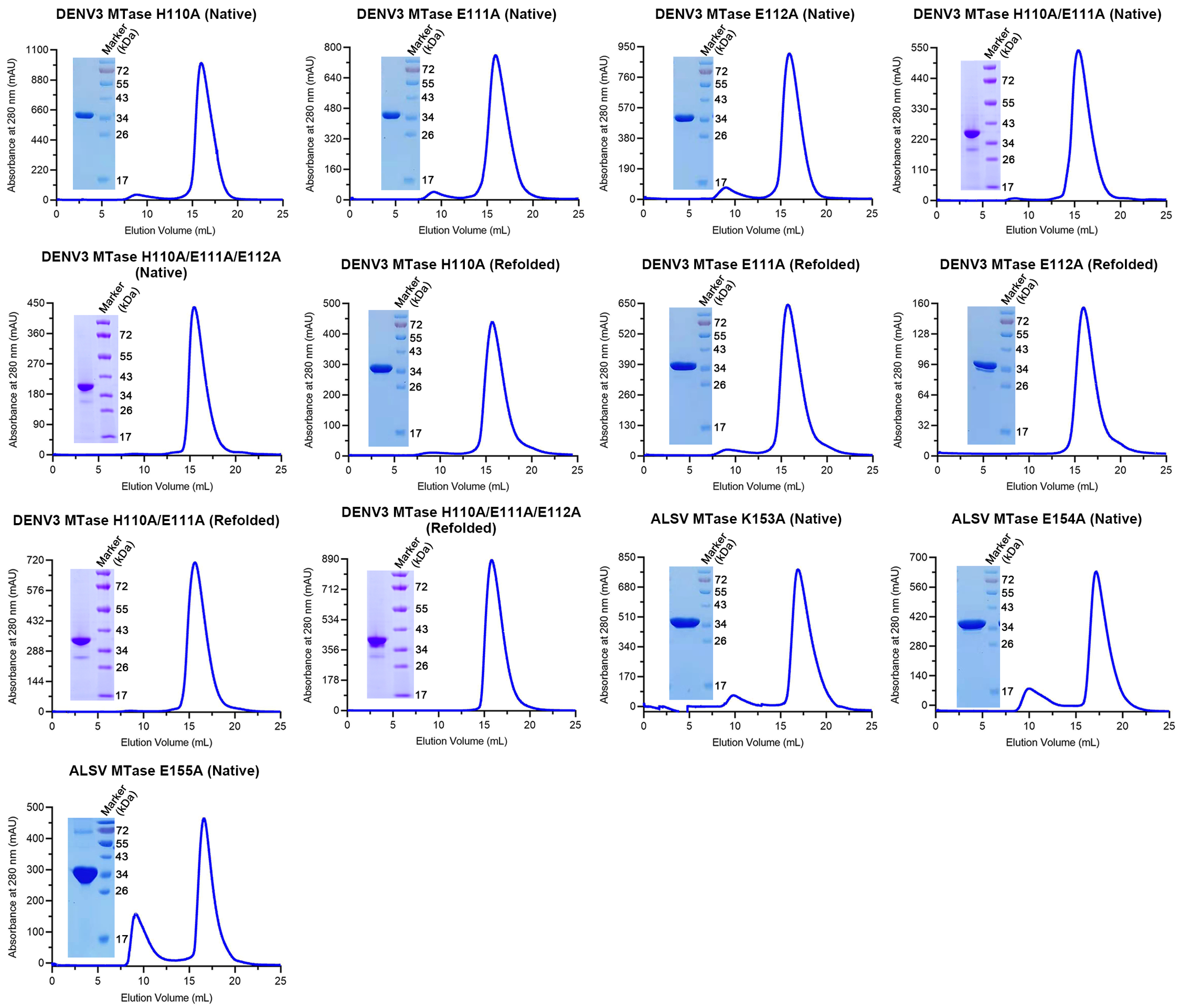

Supplement: S3 Fig — Solution behaviors of DENV3 and ALSV mutant MTase proteins on a Superdex 200 Increase 10/300 GL column. The inset figure shows the SDS-PAGE analyses of the pooled samples. (TIF) [file ppat.1011694.s003.tif]

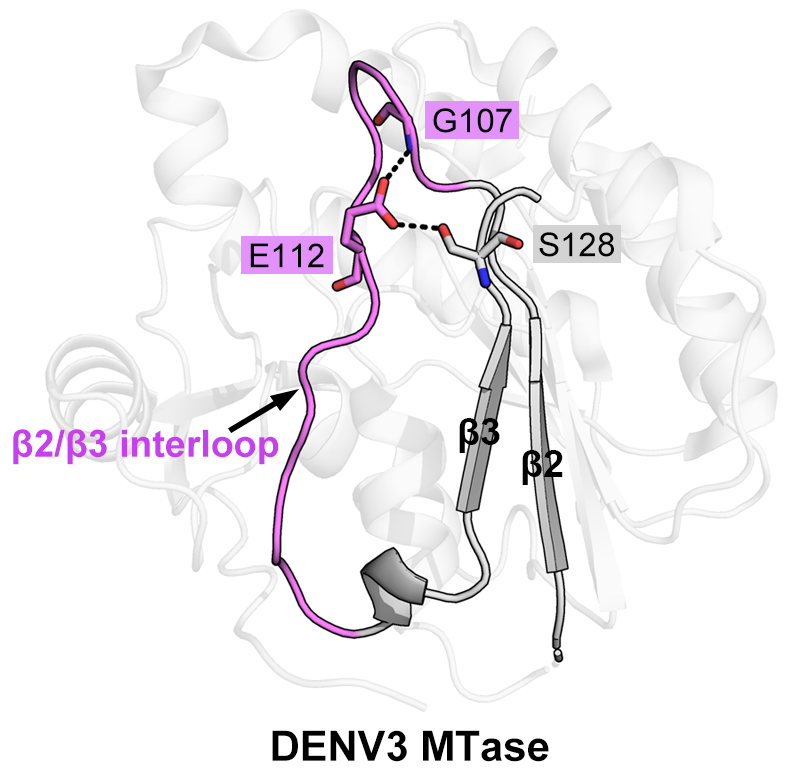

Supplement: S4 Fig — As the dashed lines indicate, the E112 residue on β2/β3 interloop could form two hydrogen bonds with G107 and S128. (TIF) [file ppat.1011694.s004.tif]

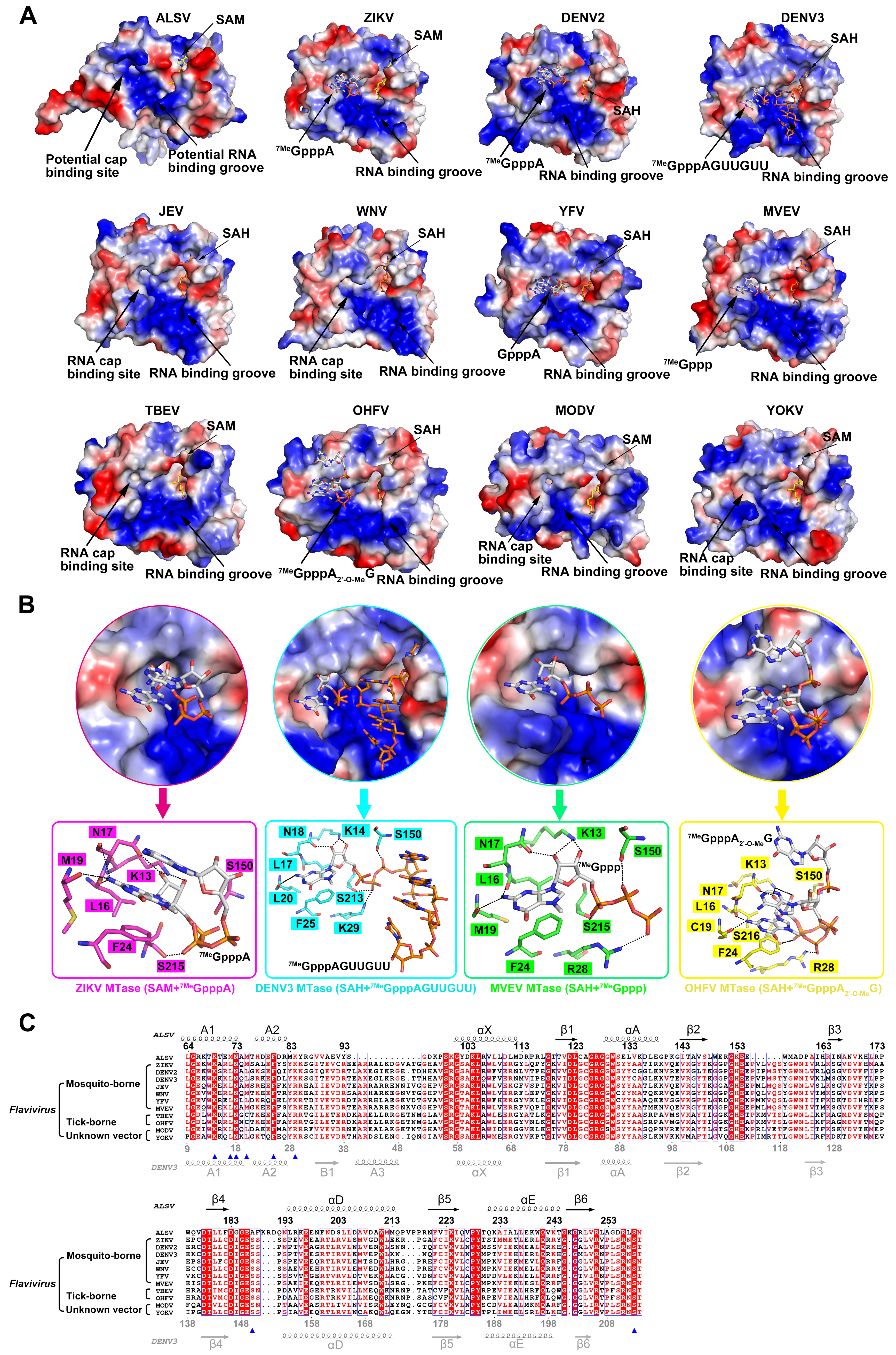

Supplement: S5 Fig — (A) Crystal structures of ALSV MTase in complex with SAM, ZIKV MTase in complex with SAM and 7MeGpppA (PDB code: 5WZ2), DENV2 MTase in complex with SAH and 7MeGpppA (PDB code: 2P3O), DENV3 MTase in complex with SAH and 7MeGpppAGUUGUU (PDB code: 5DTO), JEV MTase in complex with SAH (PDB code: 4K6M), WNV MTase in complex with SAH (PDB code: 2OY0), YFV MTase in complex with SAH and GpppA (PDB code: 3EVE), MVEV MTase in complex with SAH and 7MeGppp (PDB code: 2PX8), TBEV MTase in complex with SAM (PDB code: 7D6M), OHFV MTase in complex with SAH and 7MeGpppA2’-O-MeG (PDB code: 7V1E), MODV MTase in complex with SAM (PDB code: 2WA2), and YOKV MTase in complex with SAM (PDB code: 3GCZ). The RNA cap analogues, RNA substrates, and SAM/SAH molecules, if present in the structures, are shown and labeled. Otherwise, the RNA cap binding sites and RNA binding grooves are indicated with arrows. (B) Detailed interactions between methyltransferases of flaviviruses and RNA cap analogues. Dashed lines indicate hydrogen bonds. (C) Structure-based multiple sequence alignment highlighting the cap binding site. The secondary structural elements of ALSV MTase are labeled above the sequences, and the secondary structural elements of DENV3 MTase are labeled below the sequences. Those amino acids involved in the RNA cap binding are highlighted with blue triangles. (TIF) [file ppat.1011694.s005.tif]
