# Supplementary material for: Structural and functional basis of low-affinity SAM/SAH-binding in the conserved MTase of the multi-segmented Alongshan virus distantly related to canonical unsegmented flaviviruses
Source: PLoS Pathog. 2023 Oct 13;19(10):e1011694. doi: 10.1371/journal.ppat.1011694 (PMC10575543; doi:10.1371/journal.ppat.1011694)
Supplement: S1 Table — (DOCX) [file ppat.1011694.s006.docx]

**S1 Table. Statistics of the sequence identities among MTase proteins from Jingmenvirus group and canonical flaviviruses.**

|  | ALSV | JMTV | MGTV | GCXV | ZIKV | DENV1 | DENV2 | DENV3 | DENV4 | JEV | WNV | YFV | TBEV | USUV |
| --- | --- | --- | --- | --- | --- | --- | --- | --- | --- | --- | --- | --- | --- | --- |
| ALSV | _ | 77.70% | 78.39% | 22.38% | 19.92% | 18.56% | 17.74% | 19.32% | 17.74% | 19.85% | 18.35% | 19.17% | 18.05% | 19.48% |
| JMTV |  | _ | 94.77% | 22.03% | 18.42% | 18.18% | 19.62% | 18.56% | 16.98% | 17.60% | 17.60% | 17.67% | 17.29% | 17.98% |
| MGTV |  |  | _ | 22.73% | 19.55% | 18.18% | 18.87% | 18.56% | 18.49% | 18.35% | 17.60% | 17.67% | 18.05% | 19.10% |
| GCXV |  |  |  | _ | 21.80% | 23.11% | 22.64% | 23.48% | 22.64% | 21.35% | 21.35% | 19.17% | 20.68% | 20.97% |
| ZIKV |  |  |  |  | _ | 61.74% | 58.49% | 62.50% | 61.13% | 68.42% | 68.80% | 54.89% | 48.12% | 68.42% |
| DENV1 |  |  |  |  |  | _ | 77.65% | 83.71% | 76.89% | 58.33% | 59.47% | 53.03% | 49.24% | 58.33% |
| DENV2 |  |  |  |  |  |  | _ | 75.76% | 70.94% | 56.23% | 60.00% | 50.19% | 47.17% | 58.11% |
| DENV3 |  |  |  |  |  |  |  | _ | 72.72% | 59.47% | 60.23% | 51.89% | 50.00% | 59.10% |
| DENV4 |  |  |  |  |  |  |  |  | _ | 59.62% | 59.25% | 50.57% | 47.55% | 60.00% |
| JEV |  |  |  |  |  |  |  |  |  | _ | 77.90% | 52.26% | 48.12% | 84.27% |
| WNV |  |  |  |  |  |  |  |  |  |  | _ | 57.14% | 48.50% | 79.03% |
| YFV |  |  |  |  |  |  |  |  |  |  |  | _ | 46.24% | 52.63% |
| TBEV |  |  |  |  |  |  |  |  |  |  |  |  | _ | 46.62% |
| USUV |  |  |  |  |  |  |  |  |  |  |  |  |  | _ |
