# Supplementary material for: Structural and functional basis of low-affinity SAM/SAH-binding in the conserved MTase of the multi-segmented Alongshan virus distantly related to canonical unsegmented flaviviruses
Source: PLoS Pathog. 2023 Oct 13;19(10):e1011694. doi: 10.1371/journal.ppat.1011694 (PMC10575543; doi:10.1371/journal.ppat.1011694)
Supplement: S2 Table — (DOCX) [file ppat.1011694.s007.docx]

**S2 Table. Data collection and structure refinement statistics.**

|  | **ALSV MTase/**  **SeMet derivative** | **ALSV MTase/**  **apo form** | **ALSV MTase/SAM complex** | **ALSV MTase/SAH complex** | **ALSV MTase/SIN complex** |
| --- | --- | --- | --- | --- | --- |
| **Data collection** |  |  |  |  |  |
| Space group | *P*42212 | *P*42212 | *P*42212 | *C*2221 | *P*42212 |
| Cell dimensions |  |  |  |  |  |
| *a*, *b*, *c* (Å) | 121.01, 121.01, 89.39 | 120.45, 120.45, 88.12 | 119.57, 119.57, 87.99 | 90.84, 170.51, 172.51 | 120.18, 120.18, 88.42 |
| *α*, *β*, *γ* (°) | 90, 90, 90 | 90, 90, 90 | 90, 90, 90 | 90, 90, 90 | 90, 90, 90 |
| Wavelength (Å) | 0.9785 | 0.9785 | 0.9785 | 0.9785 | 0.9785 |
| Resolution (Å) | 50.00-2.70 (2.80-2.70) | 50.00-2.50 (2.59-2.50) | 50.00-2.30 (2.38-2.30) | 50.00-2.10 (2.18-2.10) | 50.00-2.00 (2.07-2.00) |
| *R*_merge_ | 0.100 (0.718) | 0.141 (0.740) | 0.129 (0.966) | 0.134 (0.409) | 0.124 (0.891) |
| I/σI | 41.50 (6.50) | 15.70 (2.80) | 11.53 (1.53) | 13.30 (5.20) | 23.63 (3.29) |
| Completeness (%) | 100.0 (100.0) | 100.0 (100.0) | 99.7 (99.8) | 100.0 (100.0) | 100.0 (100.0) |
| Redundancy | 18.8 (18.4) | 7.3 (7.1) | 9.5 (9.2) | 6.2 (6.4) | 21.7 (20.6) |
| Total reflections | 354029 | 168402 | 275349 | 482543 | 951351 |
| Unique reflections | 18842 | 23073 | 28864 | 77683 | 43931 |
| **Refinement** |  | |  |  |  |
| Resolution (Å) |  | 49.72-2.50 | 33.16-2.30 | 48.18-2.10 | 42.49-2.01 |
| *R*_work_/*R*_free_ |  | 0.209 | 0.192 | 0.210 | 0.198 |
|  |  | 0.258 | 0.241 | 0.236 | 0.226 |
| **No. of atoms** |  | |  |  |  |
| Protein |  | 3808 | 4014 | 7849 | 3975 |
| Ligand |  | 0 | 54 | 104 | 54 |
| Water |  | 37 | 179 | 779 | 330 |
| ***B*-factors (Å^2^)** |  | |  |  |  |
| Protein |  | 55.2 | 43.9 | 26.9 | 31.5 |
| Ligand |  | - | 46.6 | 29.0 | 34.9 |
| Water |  | 54.5 | 45.0 | 33.9 | 38.7 |
| **r.m.s.d.** |  | |  |  |  |
| Bond lengths (Å) |  | 0.008 | 0.008 | 0.008 | 0.008 |
| Bond angles (°) |  | 0.986 | 1.048 | 1.037 | 1.127 |
| **Ramachandran plot (%)** |  |  |  |  |  |
| Favored region |  | 96.72 | 97.53 | 97.36 | 97.71 |
| Allowed region |  | 3.28 | 2.27 | 2.64 | 2.29 |
| Outlier region |  | 0 | 0.21 | 0 | 0 |
| **PDB code** |  | 8GY4 | 8GY9 | 8GYB | 8GYA |

A single crystal was used to collect the data.

Values in parentheses are for the highest-resolution shell.
